# Cortisol levels and perceived stress in emergency call operators

**DOI:** 10.3389/fpubh.2025.1539516

**Published:** 2025-06-03

**Authors:** Kinga Kulczycka, Beata Chilimoniuk, Anna Rymuszka, Ewa Stychno, Agnieszka Bałanda-Bałdyga, Klaudia Pałucka

**Affiliations:** ^1^Faculty of Medicine, Institute of Health Sciences, The John Paul II Catholic University of Lublin, Lublin, Poland; ^2^Independent Laboratory of Medical Emergency Procedures and Specialized Rescue Services, Department of Emergency Medical Services, Faculty of Medicine, Medical University of Lublin, Lublin, Poland; ^3^Department of Animal Physiology and Toxicology, Faculty of Medicine, Institute of Biological Sciences, The John Paul II Catholic University of Lublin, Lublin, Poland; ^4^Department of Integrated Medical Care, Faculty of Medicine, Collegium Medicum, Cardinal Stefan Wyszyński University, Warsaw, Masovian, Poland

**Keywords:** cortisol, stress, work environment, emergency call operator, employee, well-being

## Abstract

**Introduction:**

Emergency medical dispatchers are required to provide support to the caller and organize help at the scene, frequently facing demanding situations where assistance decisions must be made promptly. The aim of this study is to assess the level of stress experienced by medical dispatchers and emergency call operators, in relation to their well-being and physical health symptoms.

**Materials and methods:**

A study was conducted in 2023 involving 23 healthy individuals employed as emergency medical dispatchers and emergency call operators, aged 26 to 65, from the Lublin Voivodeship. Data was collected based on continuous observation conducted over a 12-h day shift, utilizing the JAWS and VAS questionnaires and assessing every 2 h the following: salivary cortisol levels, heart rate, and blood pressure.

**Results:**

The employee’s level of arousal correlated with cortisol levels and significantly decreased during working hours, B = −13.87, SE = 5.16, *p* = 0.009. Among women, there was an increase in average heart rate during subsequent work hours, B = 47.4, SE = 22.0, *p* = 0.035. At the end of the workday, longer emergency caller interactions correlated with lower employee heart rates, B = −0.57, SE = 0.28, *p* = 0.046. Systolic blood pressure significantly increased with a rise in subjective stress assessment, but only during the first 2 h of work, B = 16.20, SE = 5.63, *p* = 0.005. Diastolic pressure depended on the employee group; among medical dispatchers, diastolic pressure values were higher at the beginning of the shift compared to the end, B = −40.2, SE = 23.9, *p* = 0.098, while an opposite trend with increased diastolic pressure was observed among 112 caLL operators.

**Conclusion:**

The cortisol profile is typical in the group of study participants. Attitude toward work correlates with physiological stress parameters. Longer conversations with an emergency caller during the final hours of work lead to a reduction in employees’ heart rates. Women exhibit an increase in heart rate as work progresses. Systolic blood pressure reflects subjective stress assessment during the first 2 h of work. Blood pressure values indicate a higher stress level in the 112 caLL operators group.

## 1 Introduction

In the literature addressing the phenomenon of workplace stress, there is significant interest in the relationship between the work environment and employee well-being. Both biological and social factors influence the perception of stress. Personality traits such as neuroticism, extraversion, and openness to experiences are also important (1, 2). The perception of stress is influenced by individual life experiences, social support, and a healthy lifestyle (3–5). Therefore, if the job aligns with personal passions, facilitates personal growth, and the working conditions are evaluated positively, employees generally maintain a good sense of well-being. On the other hand, a lack of well-being can mark the beginning of dissatisfaction, fatigue, and burnout. American psychologist P. Warr developed the vitamin model of employee well-being (6–9). In this model, a group of factors that can be harmful when present in excess has been identified, including workload and personal control (job autonomy), job variety, clarity of expectations and evaluations, the ability to form connections, and externally imposed goals. The second group of factors, which promote positive job assessments, includes supportive supervision, financial compensation, high social status, organizational ethics, physical job security, and career prospects. Human resources often provide the competitive advantage of an organization, making it essential to understand employee well-being, assess satisfaction, and evaluate well-being to enable interventions that improve factors contributing to positive work experiences. Therefore, researchers evaluating employee well-being increasingly focus on both emotional and environmental factors (10). The concept of stress, in everyday language, is often associated with a state of nervous tension, which is a response to negative psychological or physical stimuli (11). However, in scientific approaches, it is explained and defined differently (12). According to the definition presented by H. Selye, stress is described as “the nonspecific response of the body to any demand placed on it” (13). It can be divided into two groups through the introduction of the concepts of distress or bad stress referring to overload stress leading to illness, and good stress – eustress, which is explained as a state of complete satisfaction while maintaining an optimal level of mobilizing stress (14). According to H. Selye, the most powerful stressors include:

- psychological tension,
- a sense of uncertainty,
- failures,
- meaninglessness (15).

Some professions require performing repetitive tasks for long periods of time while maintaining a high level of concentration, which can lead to increased levels of occupational stress.

The work environment for individuals handling emergency calls and notifications of incidents is perceived as highly stressful (16). The 112 emergency line operators and medical emergency dispatchers both play critical roles in handling emergency situations, but their functions differ in terms of the tasks they perform. 112 emergency line operators are the first point of contact when someone calls the emergency services (112 is the emergency number in many European countries). Operators handling the 112 emergency number are trained to quickly assess situations and direct the appropriate services to the scene of the incident. They receive calls from individuals in life-threatening or health-threatening situations to request assistance from emergency services, such as the police, fire department or ambulance (17).

A medical emergency dispatcher specifically handles calls related to medical emergencies. After receiving information (either directly or through an operator), they dispatch paramedics or emergency medical teams to the scene of the incident. They also provide critical instructions to the caller or first responders on how to manage the medical situation until help arrives. Emergency medical dispatching is a highly intricate process, and its execution is recognized to influence patient outcomes. This process involves answering calls, assessing the severity of the situation (triage), prioritizing available pre-hospital resources, and offering guidance and instructions to those calling for help (18). Emergency number 112 operators at Emergency Notification Centers, similarly to medical dispatchers, are tasked with gathering as much useful information as possible from callers to provide appropriate assistance and forwarding the report to the relevant emergency services. The assessment of stress levels experienced during professional duties, utilizing biological samples, emphasizes cortisol as the most widely recognized stress marker within the scientific community. Cortisol is a product of the hypothalamic–pituitary–adrenal (HPA) axis, which plays a crucial role in regulating the body’s biological systems—from metabolism to immune function. Research analysis shows that cortisol levels rise in response to a stressor, which may include various physical and emotional factors. In this study, the cortisol levels of emergency call operators (999 and 112) were measured to assess their response to stress. Research conducted on various groups of individuals increasingly focuses on assessing the diurnal variability of cortisol levels, examining the influences and consequences of individual differences in the daily (circadian) rhythm of cortisol (19, 20). Cortisol profile interpretation is generally derived from the comparison between morning and evening measurements or the overall study duration and its boundary samples. Furthermore, regression analysis or multilevel growth curve modeling can be utilized to predict daily fluctuations in cortisol levels based on the time of day, with individualized measurements. Cortisol’s primary effect on the circulatory system is the increased tension in peripheral blood vessel walls, causing arterial constriction, which leads to elevated total vascular resistance and subsequent changes in blood pressure. High cortisol response to stress increases the likelihood of developing hypertension and the progression of arterial calcification (21). In high-risk professions, recurrent stressful situations are likely to adversely impact both the physical health and overall well-being of emergency service personnel. Consequently, parameters such as blood pressure and heart rate provide valuable information regarding the body’s response to stress (22, 23).

The study was conducted among emergency call operators and medical dispatchers. Operators of emergency numbers 999 and 112 are tasked with providing assistance to callers and coordinating appropriate aid at the incident scene. They often face demanding situations where decisions regarding the provision of help must be made swiftly. Callers must be provided with clear instructions on responding to life-threatening medical emergencies and be accurately understood, while operators must collect critical information about the incident and make prompt, informed decisions. Emergency calls and incident reports often involve a wide range of adverse events related to losses such as property, health, dignity, peace, and security. Although not directly involved in traumatic events, operators and medical dispatchers are responsible for listening, reacting, and intervening in response to a wide range of incidents (22, 23).

There is a lack of detailed studies in the available literature regarding the impact of the work environment and health status of emergency call operators and medical dispatchers. The literature reviews have focused on the sources and consequences of occupational stress experienced by paramedics and other ambulance personnel. The work of these professionals is demanding and stressful, which can lead to various health problems. No published review has explored the sources of stress impacting psychological health for those working in an Emergency Dispatch Center. In the literature, the importance of conducting research on stress and its consequences for the psycho-physical health of medical dispatchers and emergency call operators is emphasized (24, 25).

The aim of the study was to examine the extent to which stress levels, measured using various physiological indicators (cortisol levels, heart rate, blood pressure), differ between 112 emergency line operators and emergency dispatchers, both in terms of overall levels and dynamics over the course of a 12-h work shift. Based on previous studies and the literature on the subject, the hypotheses to be tested in this study are presented below. Hypotheses:

1. Individuals experiencing work-related stress exhibit greater fluctuations in cortisol levels compared to individuals with low levels of stress.
2. Emergency call operators have a distinct cortisol profile in saliva compared to individuals working fixed shifts.
3. The duration of telephone calls correlates with cortisol levels.
4. Higher subjective feelings of stress correlate with higher systolic blood pressure.
5. Women experiencing work-related stress have higher heart rates compared to men.
6. High levels of stress and significant emotional arousal lead to an increase in diastolic blood pressure.
7. Chronic exposure to high cortisol levels leads to the development of health problems.

## 2 Materials and methods

### 2.1 Methods of data collection

Participants were required to complete a questionnaire, which helped establish eligibility criteria by gathering information to determine whether individuals met the characteristics needed for inclusion in the study. The questions focused on the health status and health behaviors of the candidates.

After preliminary qualification, participants were asked to complete the Job-related Affective Well-being Scale (JAWS) questionnaire to assess their well-being at work. The JAWS questionnaire is used to analyze the relationship between work and employee well-being and has been successfully implemented in many countries (26). It is a 30-item scale that describes respondents’ emotional reactions to their job. The scale is based on a two-dimensional circumplex model where emotions are positioned on a continuous circle. This circumplex space is defined by two opposing dimensions: pleasure and arousal. The pleasure-displeasure dimension reflects the emotional valence, while the arousal dimension, ranging from sleep to high arousal, indicates the activation potential of emotions. Each emotional state can be identified by its specific location within this space (27).

The questionnaire consists of a series of statements describing various feelings related to work. For each statement, respondents had the option to select one of several possible answers (never, rarely, sometimes, often, very often), indicating the frequency with which they experienced specific work-related emotions over the past 30 days. The following concepts are distinguished in the questionnaire: Low Pleasure, High Arousal (LPHA)—this is a combination of low pleasure (e.g., negative emotions such as anger) and high arousal (e.g., intense activity, excitement); Low Pleasure, Low Arousal (LPLA)—this is a combination of low pleasure and low arousal (e.g., apathy, fatigue); High Pleasure, High Arousal (HPHA)—this combination is characterized by high pleasure and high arousal (e.g., joy, excitement); High Pleasure Low Arousal (HPLA)—it refers to emotions that are characterized by a high level of pleasure but a low level of arousal (positive emotions in a calm, relaxed manner, without intense activity) (26–28).

Additionally, the survey included questions to assess stress levels using a Visual Analog Scale (VAS) (29), which is a horizontal, uncalibrated line measuring 100 mm in length, ranging from very low (0) to very high (10). To collect demographic data, the questionnaire was supplemented with demographic questions.

A day-in-the-life photography method was used for data collection. It involved monitoring of the employee, measuring time, and recording all events occurring in the worksheet. Direct observation techniques were employed, which included recording activities (or inactivity) at the workplace. Snapshot observations at specific intervals were conducted during a 12-h day shift, with measurements taken every 2 h to assess salivary cortisol levels, heart rate, and blood pressure (30).

### 2.2 Participants

This study included only healthy individuals employed as medical dispatchers, consisting of 13 participants (2 females and 11 males) and 10 emergency call operators (8 females and 2 males). All participants were well-rested and had slept adequately the previous night. The participants’ ages ranged from 26 to 65 years, with an average age of 37.23 ± SD 9.50 for medical dispatchers and 35.80 ± SD 6.80 for emergency operators. Each participant provided written informed consent after receiving detailed information about the research project. The study was conducted in accordance with ethical standards, and ethical approval was granted by the Institutional Review Board (IRB; Decision No. 9/2024 of April 3, 2024).

### 2.3 Exclusion criteria

The following exclusion criteria were applied: chronic alcoholics—for over a year; individuals with mental disorders (mainly depression) undergoing treatment; women using a combination of estrogens and progestogens—during treatment; pregnant women; individuals receiving steroid treatment—within 3 months after completion of treatment; study participants presenting with fever or infection on the day of sample collection; highly athletic individuals—training regularly at least 3 times a week and with high intensity; individuals with endocrine disorders (such as adrenal disease, Cushing’s disease, Addison’s disease with low cortisol levels, pituitary gland dysfunction, hyperthyroidism); individuals suffering from diabetes or anorexia. Based on the proposed criteria, 7 individuals were excluded from the study.

### 2.4 Measurement of physiological parameters and cortisol

**Figure 1:**
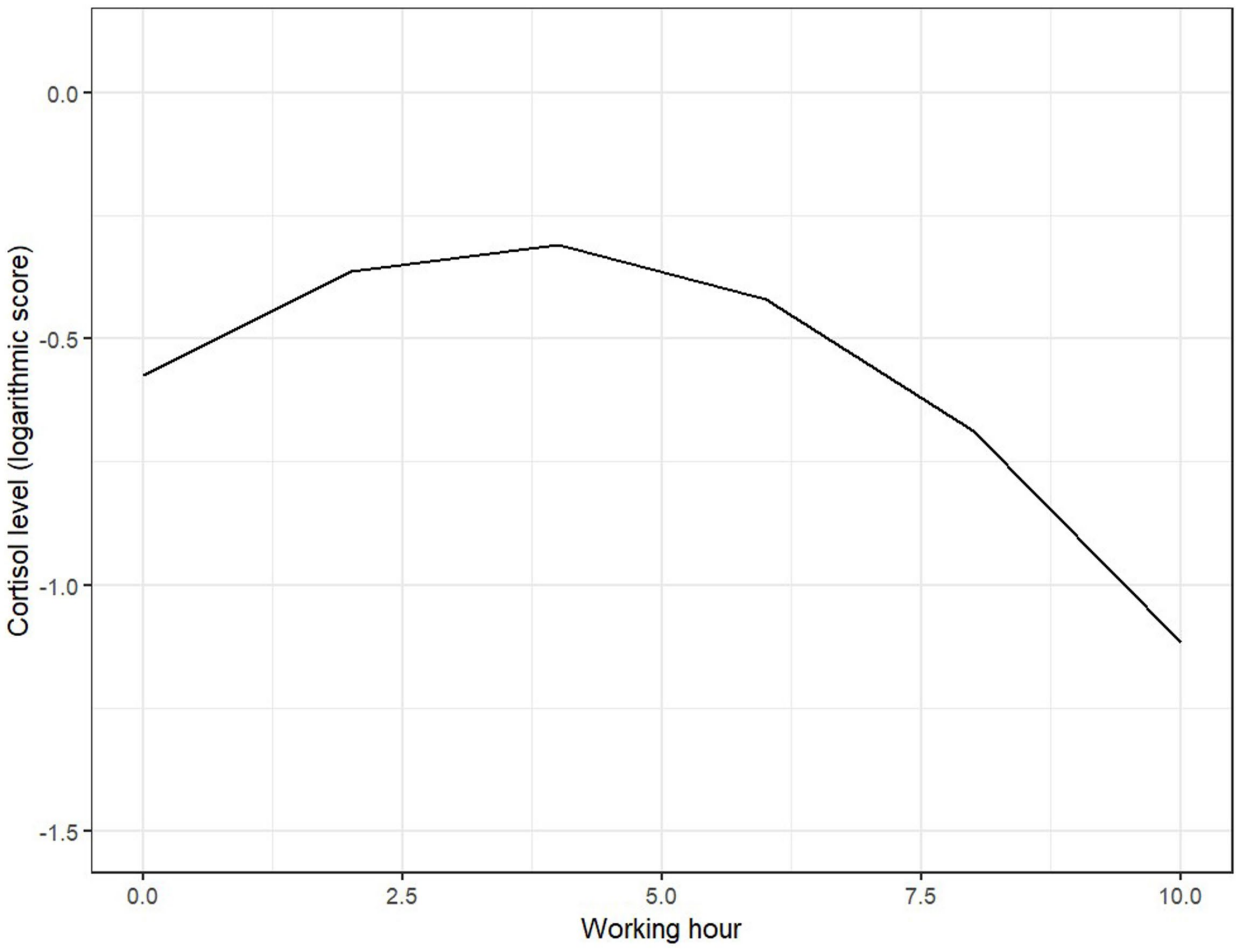
Predicted values of cortisol log.

Measurements of cortisol were systematically conducted during 12-h day shifts, commencing at 7:00 AM and continuing at two-hour intervals until 7:00 PM. Cortisol levels in saliva exhibit a cyclical secretion pattern, peaking approximately 30 min after waking. The average cortisol concentration in saliva ranges from 3.31 to 40.55 nmol/L, with the highest values occurring within the first 3 h post-awakening, and the lowest levels observed during the evening, approximately half of the morning values. Saliva samples were collected using a Salivette, following the manufacturer’s instructions. They were stored in a refrigerator at 4°C and subsequently frozen at −80°C until analysis. Participants were instructed not to eat, drink, smoke, chew gum, or brush their teeth for 30 min prior to saliva collection. Cortisol levels in saliva were measured using the ELISA method, according to the manufacturer’s instructions (Figure 1).

In addition to collecting saliva samples every 2 h, measurements of blood pressure and heart rate were also taken from the study participants. Heart rate and blood pressure were measured using a wrist blood pressure monitor that displayed the parameter readings. Normal blood pressure values were defined as threshold blood pressure of 139/89 mmHg, and heart rate was considered normal at 60–100 beats per minute. During the physiological parameter assessments, respondents provided a subjective evaluation of their stress levels using a Visual Analog Scale (VAS). Observers participating in the study recorded the number of incoming calls, their duration, the waiting time for connections, and the time allocated for breaks in the work of the employees participating in the study on that day. The obtained information underwent a comprehensive statistical analysis. For each variable, the mean and standard deviation were calculated.

### 2.5 Data analysis

Data analysis was performed using the lme4 package in R (31). The data were examined for outliers and the distribution of data points using histograms with a normal distribution curve and skewness and kurtosis analysis. To determine the relationships between variables, a Student’s t-test and Pearson correlation were applied, with a significance level of 0.05.

To test hypotheses regarding differences in stress levels during the day shift between 112 emergency operators and medical dispatchers, expressed in fluctuations of physiological stress indicators experienced during work, we conducted four developmental curve analyses using multilevel models. In each of these models, the main variable was one of the physiological stress indicators: cortisol level, heart rate, systolic blood pressure, and diastolic blood pressure. In each model, the main predictor was time, measured in two-hour intervals, from 7:00 AM (with the first measurement at 9:00 AM, *t* = 0) to 7:00 PM (*t* = 10). We introduced two orthogonal time-related effects into the model: linear and nonlinear (quadratic). To examine whether the level of the physiological stress indicator changed over time differently depending on the group, we also included an interaction between group and time in the model. Changes in physiological stress indicators during work could also depend on a range of other variables, such as gender, age, VAS, body weight, height, work experience (years of service), client contact time, subjectively perceived stress, or individual differences in the tendency to experience certain emotions (positive vs. negative, and high arousal vs. low arousal) related to stress. Descriptive statistics are presented in Table 1. In preliminary analyses (*t-*tests; Table 2), no significant differences were observed between the groups of operators and medical dispatchers with regard to the aforementioned covariates. Therefore, interactions between time and all these covariates were also included in each of the four models. In each of the four models, the intercept could vary by individual (random effect). Before entering the variables into the model, they were centered around the grand mean for all participants. The models were estimated using the Restricted Maximum Likelihood (REML) method. Due to the non-normal distribution of cortisol levels, the results for this variable were log-transformed prior to analysis.

**Table 1:** Descriptive statistics of each variable.

Row	n	Mean	Sd	Median	Min	Max	Range	Skew	Kurtosis
Age	23	36.61	8.28	35.00	26.00	65.00	39.00	1.58	3.46
Total Experience	23	12.70	9.01	10.00	0.08	45.00	44.92	1.86	4.54
Current Experience	23	5.70	4.21	6.00	0.08	15.00	14.92	0.45	−0.95
Height	23	176.04	8.84	174.00	158.00	195.00	37.00	0.41	−0.31
Weight	23	79.09	16.67	78.00	47.00	107.00	60.00	−0.02	−0.80
Availability	23	13.83	3.60	14.00	6.00	20.00	14.00	−0.33	−0.57
Expectation	23	17.65	4.25	18.00	9.00	27.00	18.00	0.01	−0.40
Break	23	5.78	2.59	5.00	2.00	13.00	11.00	0.81	0.43
Requirements	23	3.90	0.44	4.00	3.12	4.50	1.38	−0.11	−1.35
Control	23	3.58	0.49	3.67	2.50	4.33	1.83	−0.53	−0.73
Supervisor Support	23	3.82	0.64	3.80	2.60	4.60	2.00	−0.49	−0.88
Co-worker Support	23	4.13	0.72	4.00	2.00	5.00	3.00	−0.96	1.03
Relationships	23	4.02	0.66	4.25	2.25	5.00	2.75	−0.88	0.44
Role	23	4.67	0.36	4.80	4.00	5.00	1.00	−0.54	−1.32
Change	22	3.91	0.93	4.00	1.00	5.00	4.00	−1.23	1.90
Job Stress	23	3.33	0.26	3.31	2.57	3.66	1.09	−1.18	1.19
HPHA	22	2.82	0.74	2.80	1.60	4.00	2.40	0.03	−1.39
HPLA	22	2.92	0.59	2.80	1.80	4.00	2.20	0.22	−0.91
LPHA	22	1.93	0.65	1.90	1.00	3.40	2.40	0.37	−0.64
LPLA	22	1.96	0.67	2.00	1.00	3.20	2.20	0.15	−1.02
Heart Rate (mean)	23	68.26	8.79	66.83	55.17	87.83	32.67	0.56	−0.57
Systolic Pressure (mean)	23	129.07	12.73	130.50	99.67	152.50	52.83	−0.64	−0.07
Diastolic Pressure (mean)	23	78.72	8.76	79.83	63.83	96.00	32.17	0.06	−0.97
Service Time (mean)	23	1289.22	615.14	1451.17	98.00	2191.50	2093.50	−0.82	−0.40
Cortisol Log (mean)	21	−0.44	0.37	−0.56	−0.99	0.53	1.51	0.88	0.24

**Table 2:** Differences between groups (*t*-tests).

Variable	Group 1	Group 2	n1	n2	Statistic	df	*p*-value	p.adj	p.adj.significance
Disposition	Dispatcher	Operator	13	10	0.72	19.19	0.48	0.60	ns
HPHA	Dispatcher	Operator	13	10	1.04	19.98	0.31	0.60	ns
HPLA	Dispatcher	Operator	13	10	−0.26	19.93	0.80	0.80	ns
Control	Dispatcher	Operator	13	10	−0.45	20.09	0.66	0.75	ns
LPHA	Dispatcher	Operator	13	10	−0.98	17.04	0.34	0.60	ns
LPLA	Dispatcher	Operator	13	10	−2.03	18.38	0.06	0.57	ns
Weight	Dispatcher	Operator	13	10	0.72	16.66	0.48	0.60	ns
Expectation	Dispatcher	Operator	13	10	1.49	20.17	0.15	0.60	ns
Break	Dispatcher	Operator	13	10	−1.75	11.83	0.10	0.60	ns
Relationships	Dispatcher	Operator	13	10	0.29	18.33	0.78	0.80	ns
Role	Dispatcher	Operator	13	10	2.29	14.74	0.04	0.57	ns
Total Experience	Dispatcher	Operator	13	10	0.97	20.81	0.34	0.60	ns
Current Experience	Dispatcher	Operator	13	10	−1.55	19.23	0.14	0.60	ns
Work Stress	Dispatcher	Operator	13	10	0.80	12.75	0.44	0.60	ns
Age	Dispatcher	Operator	13	10	0.42	20.93	0.68	0.75	ns
Support from Supervisor	Dispatcher	Operator	13	10	0.97	13.34	0.35	0.60	ns
Support from Colleagues	Dispatcher	Operator	13	10	0.73	16.62	0.47	0.60	ns
Demands	Dispatcher	Operator	13	10	0.73	20.08	0.47	0.60	ns
Height	Dispatcher	Operator	13	10	1.19	20.37	0.25	0.60	ns
Change	Dispatcher	Operator	13	10	1.39	13.33	0.19	0.60	ns

## 3 Results

### 3.1 Cortisol

**Figure 2:**
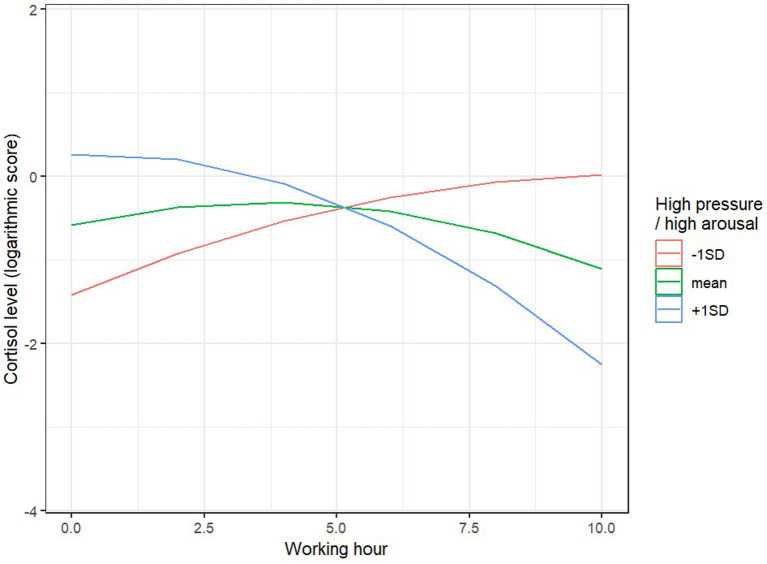
Predicted values of cortisol log and HPHA.

The fixed effects (related to predictors and interactions) explained 49% of the variance in cortisol levels during the 10-h workday (61% when accounting for random effects). We observed a significant main effect of client contact time, *F* (1, 61.34) = 4.00, *p* = 0.05: the longer the phone conversation with the caller during a given hour of work, the lower the cortisol level during that hour. We also observed two significant interaction effects between the linear time effect and HPHA, *F* (1, 61.02) = 5.94, *p* = 0.018, and LPLA, *F* (1, 61.43) = 5.04, *p* = 0.028. In individuals with higher HPHA levels (1 point above the mean), cortisol levels significantly decreased during the work hours, B = −13.87, SE = 5.16, *p* = 0.009, while for individuals with lower HPHA levels (1 point below the mean), there was a trend toward an increase in cortisol levels, B = 8.62, SE = 4.45, *p* = 0.056 (Figure 2).

**Figure 3:**
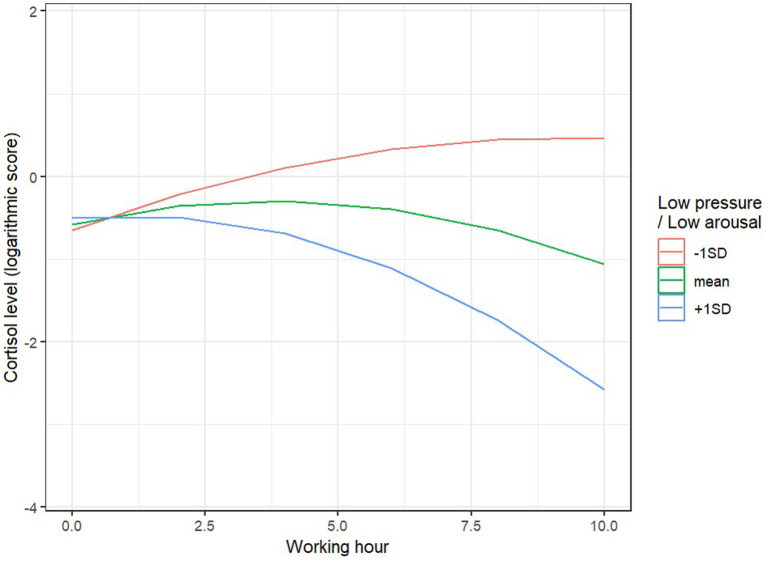
Predicted values of cortisol log and LPLA.

A similar effect was observed for the LPLA variable: in individuals with higher LPLA levels (1 point above the mean), cortisol levels significantly decreased during the work hours, B = −11.43, SE = 4.40, *p* = 0.012, while for individuals with lower LPLA levels (1 point below the mean), there was a trend toward an increase in cortisol levels, B = 78.68, SE = 4.52, *p* = 0.095 (Figure 3). The remaining main and interaction effects were not significant.

### 3.2 Heart rate

**Figure 4:**
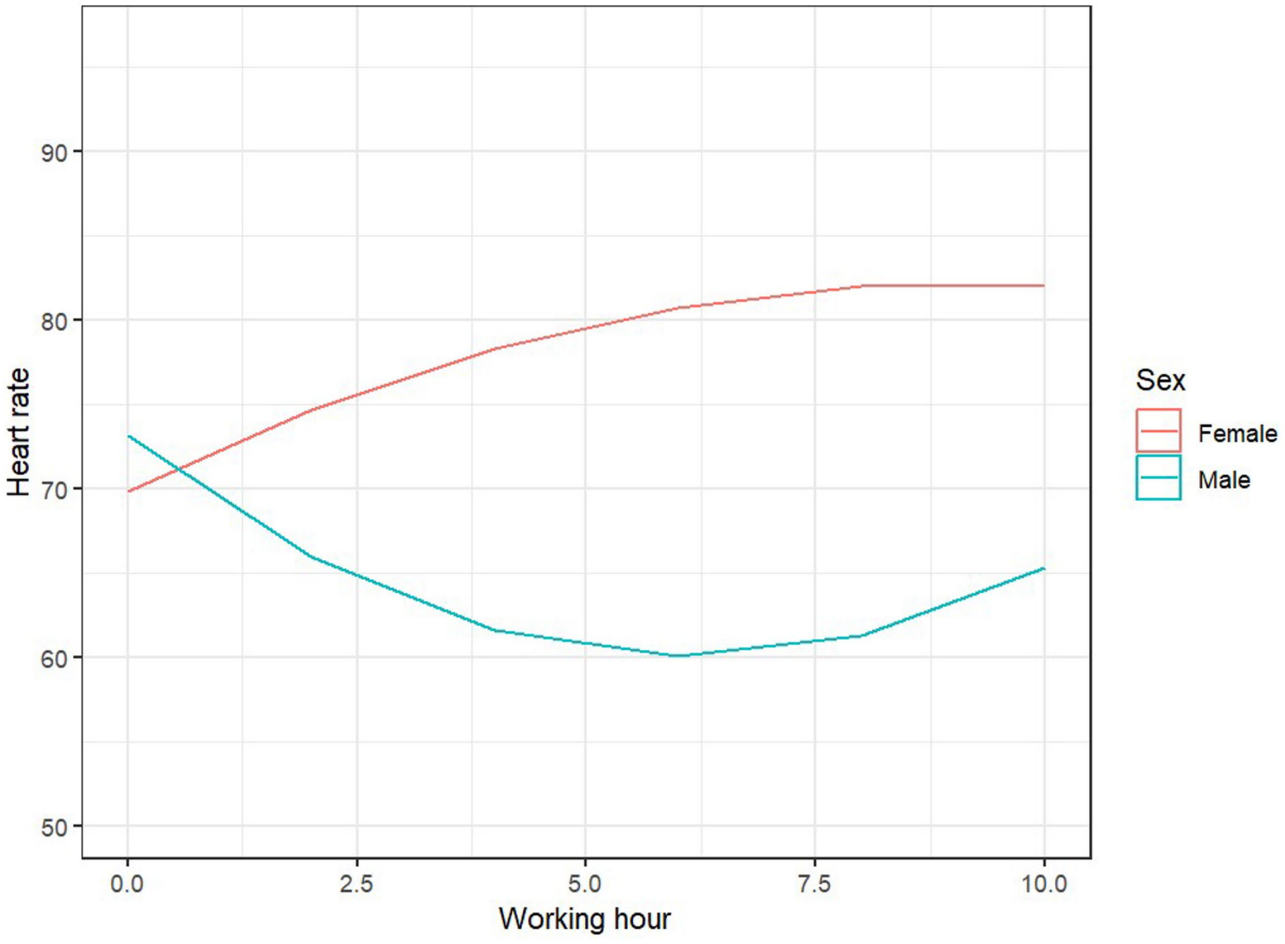
Predicted values of HR and sex.

**Figure 5:**
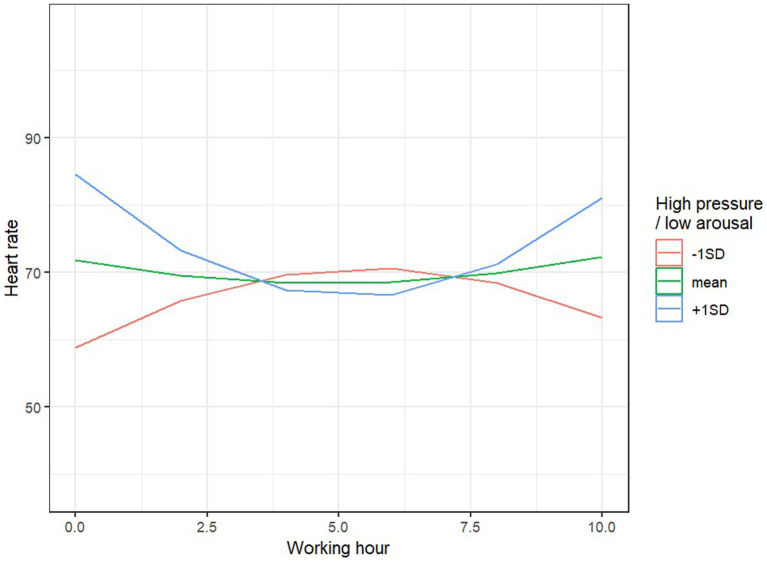
Predicted values of HR and HPLA.

The model explained 34% of the variance in heart rate during work (81% when accounting for random effects). None of the main effects were significant. However, we observed three significant interaction effects: the linear time effect with sex, *F* (1, 60.09) = 6.77, *p* = 0.012, the time of service, *F* (1, 63.23) = 4.13, *p* = 0.046, and the nonlinear time effect with HPLA, *F* (1, 60.05) = 4.75, *p* = 0.033. For women, the average heart rate significantly increased during the successive hours of work, B = 47.4, SE = 22.0, *p* = 0.035, while for men, the time effect was not significant, B = −32.7, SE = 19.9, *p* = 0.105 (Figure 4). At the beginning of the workday (the first 2 h), the length of client contact was not associated with heart rate levels, B = 0.001, SE = 0.002, *p* = 0.52; however, toward the end of the workday (the last 2 h), the longer the client contact, the lower the heart rate of the employee, B = −0.57, SE = 0.28, *p* = 0.046. Individual differences in HPLA also influenced heart rate dynamics: for individuals with low HPLA levels, heart rate initially increased to a maximum level mid-work, then decreased, B = −87, SE = 48.7, *p* = 0.079, while for individuals with high HPLA levels, the effect was reversed: heart rate initially decreased to a minimum, then increased, B = 111, SE = 45.6, *p* = 0.018 (Figure 5).

### 3.3 Systolic blood pressure

**Figure 6:**
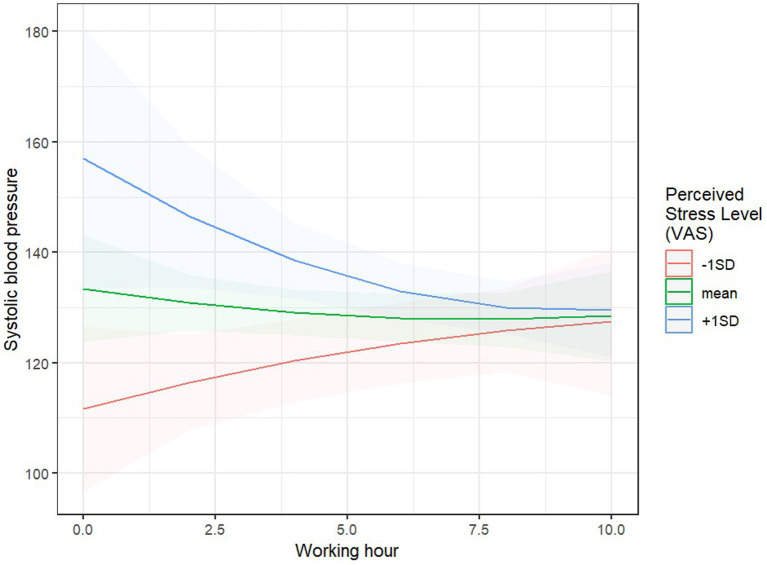
Predicted values of systolic blood pressure and perceived stress.

The model explained 45% of the variability in systolic blood pressure (60% when accounting for random effects). The main effect of the Visual Analog Scale (VAS) was significant, *F* (1, 65.99) = 9.23, *p* = 0.003: the higher the intensity of subjectively perceived stress, the higher the systolic blood pressure. However, this effect was moderated by the length of work performed: the interaction between the linear time effect and VAS was significant, *F* (1, 65.70) = 6.07, *p* = 0.016. Simple effects analysis revealed that systolic blood pressure significantly increased with higher subjective stress ratings, but only at the beginning of the work period, B = 16.20, SE = 5.63, *p* = 0.005 (after the first 2 h), while at the end of the day, no relationship between VAS and systolic blood pressure was found, B = −1.35, SE = 2.38, *p* = 0.574 (after 12 h of work; Figure 6). Additionally, significant interaction effects were observed between linear time and body weight, *F* (1, 60.69) = 4.037, *p* = 0.049, and age, *F* (1, 62.46) = 4.91, *p* = 0.030, as well as an interaction between the nonlinear time effect and LPHA, *F*(1, 61.65) = 6.49, *p* = 0.013. Systolic blood pressure significantly decreased during working hours only for younger individuals, B = −139.7, SE = 51.7, *p* = 0.009, and those with higher body weight, B = −107.5, SE = 48.5, *p* = 0.030, compared to older individuals B = 82.1, SE = 58.3, *p* = 0.164, and those with lower body weight, B = 25.5, SE = 31.0, *p* = 0.414. For individuals with high LPHA levels, systolic blood pressure during work initially decreased, then increased, B = 176, SE = 65.1, *p* = 0.009, whereas for individuals with low LPHA levels, systolic blood pressure initially increased, then decreased, B = −173, SE = 78.2, *p* = 0.030.

### 3.4 Diastolic blood pressure

The model explained 39% of the variability in diastolic blood pressure (77% when accounting for random effects). We did not observe any significant main effects. However, three significant interaction effects were identified between working time and: group, *F* (1, 60.35) = 4.33, *p* = 0.039, average all-day client service duration, *F*(1, 60.35) = 5.43, *p* = 0.023, and LPHA, *F* (1, 60.56) = 7.64, *p* = 0.008. Simple effects analysis revealed that diastolic blood pressure exhibited an increasing trend during working hours for operators, B = 50.5, SE = 26.7, *p* = 0.064, while for dispatchers, there was a tendency for it to decrease, B = −40.2, SE = 23.9, *p* = 0.098. Regardless of group membership, a lower average all-day client service time was associated with a gradual increase in diastolic blood pressure throughout the day, B = 49.8, SE = 23.5, *p* = 0.039, while for individuals with higher all-day client service times, the effect was not significant, B = −25.6, SE = 18.7, *p* = 0.174. Additionally, for individuals with high LPHA levels, diastolic blood pressure during work initially decreased, then increased, B = 108, SE = 38.1, *p* = 0.006, while for individuals with low LPHA levels, diastolic blood pressure initially increased, then decreased, B = −114, SE = 45.9, *p* = 0.016.

## 4 Discussion

Scientific research indicates that abnormal secretion of the glucocorticoid hormone cortisol, which is the end product of the hypothalamic–pituitary–adrenal (HPA) axis, is a key factor linking chronic psychosocial stress experiences to adverse health effects. It has been found that the daily rhythm of cortisol secretion in humans and its concentration in bodily fluids may only reflect individual stress experienced in the minutes or hours prior; therefore, repeated sampling is necessary (31). Saliva allows for the examination of steroid hormones in their free fraction, providing the advantage of easy sample collection. The concentration of hormones in saliva is 10 to 100 times lower than in serum but is consistent and analogous to serum concentrations. Due to the rapid transfer of these hormones, measurement in saliva is representative of blood serum concentrations (32, 33). It is also important to note that cortisol levels in saliva are independent of the rate of saliva secretion, as the diffusion of this hormone occurs very quickly (34). The method of material collection is minimally invasive, which helps avoid errors caused by stress during blood sampling for tests, thereby facilitating measurements several times a day. This is particularly useful when analyzing the kinetics of cortisol secretion and determining the daily profile of this hormone’s release. The choice of laboratory measurement methods in this study was primarily dictated by ease of use, availability, and cost-effectiveness. Saliva sampling is a simple and safe process that can be easily performed multiple times in the work environment without causing stress or requiring individuals to leave their workplace. In the case of saliva samples, the risk of contamination is lower compared to other collection methods (35, 36).

### 4.1 Cortisol secretion and stress

Based on multiple studies, a typical diurnal cortisol secretion pattern has been identified. Cortisol peaks—rising by an average of 50–60% within 30 to 45 min after awakening—then gradually decreases, reaching its lowest level around midnight during sleep (37). In the conducted study, cortisol levels rise and sustain for approximately 3 h, after which a downward trend is observed, indicating that the cortisol profile among the respondents can be characterized as typical and within normal limits. However, there are also studies indicating individual differences in daily cortisol profiles (38).

Cortisol levels respond not only to social and psychological stress (39, 40) but also to acute and chronic stress (41). Cortisol is undoubtedly a hormone associated with the organism’s emotional responses, but its levels cannot be interpreted as a direct, proportional assessment of stress (42, 43). For instance, cortisol can spike in response to perceived positive, exciting, and enjoyable experiences, such as sports competitions among adults (44, 45). To assess well-being and emotional responses, the JAWS questionnaire was used, and statistically significant correlations were observed between cortisol levels and the emotional state of respondents. For individuals with higher excitement levels, cortisol significantly decreased during working hours, whereas those with lower positive emotions and engagement showed an upward trend in cortisol levels. A similar effect was observed in individuals with higher levels of work-related discouragement, where cortisol significantly decreased during working hours, while for individuals with lower levels of discouragement or meaninglessness, an upward trend in cortisol levels was observed. This suggests the significant impact of emotional engagement on responses assessed through cortisol levels, which are interpreted as stress-related.

### 4.2 Cortisol awakening response and stress

Researchers have not been able to definitively determine the role of cortisol level increases post-awakening. Some studies suggest an association between morning cortisol spikes and physiological health and psychological well-being, particularly among individuals with high stress levels or increased work burden (46–48). However, other studies, focusing on groups with symptoms of burnout, have reported lower cortisol levels post-awakening (49). It is hypothesized that low morning cortisol levels may result from a weakened cortisol awakening response following prolonged exposure to stress (50). Sleep disturbances can alter nighttime cortisol secretion, weakening the feedback regulation mechanism of cortisol (51). Shift workers may experience fluctuations in cortisol levels, reflecting responses to daily variations in social and emotional experiences. Studies have found that cortisol levels were lower in individuals with irregular shift patterns than in those with regular shifts (52). There are also studies among nurses working night shifts that confirm significantly higher cortisol levels compared to a control group of nurses working only daytime shifts (53). Therefore, it can be concluded that shift work may alter individual cortisol patterns.

### 4.3 Heart rate and stress

Another physiological parameter used to assess stress is heart rate, as an increased heart rate may be a sign of heightened emotional arousal (54). Under stressful conditions, such as time pressure, heart rate variability (HRV) tends to decrease (55). Therefore, both heart rate and heart rate variability are considered physiological markers of stress and anxiety (56, 57). Based on the study results, it was found that women exhibited an increase in average heart rate over successive work hours, whereas in men, the effect of time was non-significant. During the first 2 h of work, the length of client interactions was not related to heart rate, but by the end of the workday, longer client interactions correlated with lower heart rate among employees. The increase in heart rate among women may be associated with greater emotional engagement in their work, and similar results were obtained when analyzing blood pressure values and work engagement. The association between occupational events and hypertension was higher in women compared to men (58). Women tend to report higher levels of perceived stress, anxiety, and tension during and after exposure to acute stress compared to men. Furthermore, possible explanations for this gender-specific pattern of response in humans range from the influence of the type of stressor (social rejection challenges as typical stressors for women, achievement stressors as typical stressors for men) to the modulating effect of hormonal fluctuations at different phases of the menstrual cycle (59).

### 4.4 Workplace stress and its impact on physiological responses

Individual differences among people who experience frustration at work influenced the dynamics of heart rate changes. In cases of low frustration, heart rate increased to a peak level mid-shift and then decreased. In contrast, individuals with high frustration showed the opposite pattern: heart rate first dropped to a minimum and then increased. The work environment has a significant relationship with cortisol levels, making the workplace a critical factor. It was found that shift work in emergency rooms elevated salivary cortisol levels (56), while another study conducted among paramedics showed no relationship between shift work and cortisol secretion (60). The workplace of emergency call operators (CPR) and medical dispatchers (DM) is described in the literature as particularly stressful due to the contact with witnesses or participants of life-threatening events and the constraints of call centers (61–63). In a study by S. Bedini et al., receiving emergency calls led to increased cortisol levels depending on the severity of the call and a cumulative effect from subsequent emergency calls (25). The authors did not attempt to evaluate the severity of the calls or classify them as either severe or mild. The professionalism of emergency call operators, their ability to provide expert assistance, and the frequency of calls—often related to tragic but repetitive situations—make it challenging to determine which calls should be classified as severe. Therefore, the quantity and duration of the calls were evaluated. The obtained study results show a relationship between call duration and cortisol levels. The longer the phone conversation with a client during a given hour, the lower the observed cortisol level. It can be speculated that shorter calls are associated with the need to make quick decisions, which in turn leads to higher cortisol levels. A situation involving longer conversation times is usually associated with the need to provide a lot of information that does not require immediate action. The caller is asked to follow instructions and/or gather necessary information. The precise transmission of information involves significant engagement, which can be viewed as a stress reducer. Similar results were found by S. Bedini, who observed an increase in cortisol levels in response to incoming emergency calls in urgent situations (*p* = 0.03), as well as an upward trend during subsequent calls requiring prompt decisions (*p* = 0.07) (20). In summary, the change in emergency call patterns poses a particular risk for dispatchers, who experience greater stress and an increase in cortisol levels. Almost every call to emergency numbers (112 or 997) involves contact with a stressed or anxious caller seeking help for themselves or someone else. The person responsible for answering the call has the critical task of quickly identifying the nature of the emergency, its severity, and the necessary resources to deploy while keeping the caller calm so that they can answer the required questions.

### 4.5 Blood pressure and subjective stress

Individual experiences of emergency call operators, as well as the disposition of their day, are key in assessing call difficulty, which is why they were asked to provide a subjective stress assessment using a Visual Analog Scale (VAS). It was found that higher levels of subjectively perceived stress led to an increase in systolic blood pressure. However, systolic blood pressure significantly increased with the rise in subjective stress assessment only during the first 2 h of work. Whereas at the end of the workday, there was no relationship between VAS and systolic blood pressure. Systolic blood pressure significantly decreased during work hours only in younger individuals and those with higher body mass, compared to older individuals and those with lower body mass. For individuals with high levels of frustration, systolic blood pressure initially decreased during the workday and then increased, whereas for individuals with low levels of frustration, systolic blood pressure initially increased and then decreased. The interaction between VAS and systolic blood pressure is intriguing. Fatigue likely plays a key role in the interaction between VAS and systolic blood pressure. The adaptive mechanism of mobilizing energy resources (allostasis) can lead to significant wear and tear on the body if it persists over time, as is the case with chronic stress (allostatic load). Consistent with these observations is a recent study: in a large population of healthy young adults, high levels of stress also led to blunted heart rate responses and additionally resulted in lower habituation over time (64).

The results of the study indicate that diastolic blood pressure levels were dependent on the employee group. Among medical dispatchers, diastolic blood pressure values were higher at the beginning of the shift compared to the end of the shift. This phenomenon can be explained by the body’s natural circadian rhythm, which regulates hormonal fluctuations to align them with daily activity patterns and support optimal functioning throughout the day. Under normal physiological conditions, cortisol levels show a significant increase within the first 30 to 45 min after waking, a phenomenon known as the Cortisol Awakening Response (CAR). This peak prepares the body for the demands of the day by mobilizing energy reserves, increasing glucose availability, and modulating immune functions. After this morning rise, cortisol levels gradually decrease throughout the day, reaching their lowest point in the early evening (65). Whereas an opposite trend, with increasing diastolic pressure, was observed among CPR operators. It can be inferred that the work process leads to an increase in stress levels among the group of 112 operators. Interesting results were also obtained from the analysis of call duration and working hours. Without visible differences between groups, a low average daily customer service time was associated with a gradual increase in diastolic blood pressure throughout the day, while for those with longer daily customer service time, the effect was non-significant. It can be speculated that quick and efficient assistance in crisis situations is a source of increased emotional tension, resulting in higher cortisol levels.

For individuals with high levels of work-related discouragement combined with high arousal (LPHA), systolic blood pressure followed a similar pattern to general systolic blood pressure trends, i.e., it initially decreased during work and then increased, while for those with low levels of LPHA, systolic blood pressure initially increased and then decreased. This result confirms an adaptive mechanism to existing work conditions: individuals who are both discouraged and highly aroused experience increasing fatigue during work hours, which manifests as an increase in diastolic pressure. In contrast, individuals who are not discouraged by work and not highly aroused experience stabilization after several hours of work, and both systolic and diastolic pressures decrease.

Assuming normal blood pressure values to be below 139/89, we can conclude that hypertension was not observed among the study participants. The average values in the groups were as follows: dispatchers 133/81, operators 122/75. Thus, the stress experienced does not appear to cause negative health effects. Other studies indicate that the prevalence of hypertension among individuals with moderate work stress was twice as high (95% CI: 1.003–4.193), and among individuals with high work stress, it was 2.87 times higher than among individuals with low work stress (95% CI: 1.142–7.194). Individuals with high work stress had an average increase in systolic blood pressure of 3.43 mmHg (95% CI: 2.02–4.84) and an average increase in diastolic blood pressure of 2.07 mmHg (95% CI: 1.17–2.97) compared to individuals with low work stress (66, 67). Numerous studies also confirm the association between hypertension and long working hours, as well as cortisol reactivity. Women exhibited a stronger association between occupational constraints and hypertension compared to men (68). There are many factors that can influence variable work engagement, such as older or middle age, affective experiences, physical activity, or circadian rhythms (69, 70). It is likely that different factors are perceived differently by each person, with some being more suitable or important for certain individuals depending on their work outcomes, as well as their family situation, personality, and priorities. For instance, a positive attitude toward parenthood and flexible work arrangements seem to be more beneficial for overall well-being and work engagement among working parents (71, 72).

Considering the specifics of the job and the factors that can lead to increased stress levels, solutions should be introduced to minimize the negative health effects caused by hypersecretion of cortisol. Elevated cortisol levels sustained over several days can lead to serious conditions, such as metabolic disorders, including type 2 diabetes, as well as depression, mental health disorders, and hypertension (73).

### 4.6 Workplace environment and support systems

The modern workplace in a new building with excellent facilities supports the effectiveness of preventive measures aimed at reducing the occurrence of high stress levels. The authors’ observations during the study show that the organizational and technical conditions are of a very high standard. Although employees spend many hours at work, they are provided with the conditions to prepare and eat meals, as well as comfortable spaces for rest and relaxation. Relationships within the team often extend beyond the formal work environment, and this is considered an added value, highly appreciated by the respondents. Good cooperation, positive relationships, and constructive support from the management are also of great importance. Medical dispatchers have the opportunity to regularly collaborate with a psychologist who is well-acquainted with the specifics of their work and is present at the workplace. There are many studies about emergency operators, but most of them focus only on certain aspects of this work, and there is a lack of proper attention to psychological factors. There is a need for further scientific inquiry into cortisol levels and perceived stress in emergency call operators (74).

It should also be noted that healthcare workers are at high risk of experiencing a lack of respect while performing their duties. Situations, such as patients addressing healthcare workers inappropriately, patients and consultants having unrealistic perceptions of nurses’ workload, or unfair behavior from some managers and colleagues are examples of mistreatment in these environments (75). Moreover, emotional experiences function as fundamental mechanisms that connect the work domain with the non-work domain. Significant events in the workplace trigger emotional reactions in employees and can influence various workplace behaviors. Therefore, mistreatment is an emotionally charged event that can generate negative affective reactions and have serious consequences for both well-being and performance (76).

### 4.7 Conclusion

The conducted research allows for the conclusion that, despite the stress associated with emergency number operators’ positions and shift work, environmental factors at the workplace do not have a negative impact on employees’ health. Monitoring and analyzing individual reactions to existing hazards will enable the implementation of actions that allow employees to carry out their duties without compromising their health. The work environment, both physically and psychologically, is a critical element that should be under constant control, which will facilitate the implementation of best practices that promote health maintenance. Identifying stressful events can be crucial for safety practices.

### 4.8 Limitations of the study

The study has several limitations. First, it is a quasi-experimental study, which does not allow for the establishment of causal relationships. Second, the small sample size may have contributed to some statistically insignificant results. It would be advisable to continue the research on a larger sample group. Additionally, there was also a significant disparity between the genders of the study participants. It would be beneficial to select a study group with equal representation of both genders, as this could provide more reliable results, considering the significant differences in physiological responses to stress between women and men. The exclusion criterion for women using oral contraceptives further exacerbates this selection bias by systematically excluding a substantial portion of the female population. While this exclusion is crucial for controlling the potential confounding effects of hormonal influences on the study outcome, it limits the findings’ generalizability to the broader female population. The subjective assessment of working conditions (the organization of work and the working conditions) was also not investigated. The organizational level and favorable working conditions in the workplace can influence different stress responses.

## Data Availability

The original contributions presented in the study are included in the article/supplementary material, further inquiries can be directed to the corresponding author.
